# HIV-serodifferent couples’ perspectives and practices regarding HIV prevention strategies: A mixed methods study

**DOI:** 10.1371/journal.pgph.0000620

**Published:** 2022-08-17

**Authors:** James M. McMahon, Janie Simmons, Amy Braksmajer, Natalie LeBlanc

**Affiliations:** 1 School of Nursing, University of Rochester Medical Center, Rochester, New York, United States of America; 2 School of Global Public Health, New York University, New York, New York, United States of America; 3 Department of Sociology, State University of New York at Geneseo, Geneseo, New York, United States of America; University of Manitoba College of Medicine: University of Manitoba Max Rady College of Medicine, CANADA

## Abstract

A substantial proportion of heterosexually acquired HIV infections in the U.S. occur between partners in primary relationships characterized by mixed HIV status. The U.S. Centers for Disease Control and Prevention have issued guidelines prioritizing HIV-serodifferent couples for primary HIV prevention, including treatment-as-prevention and pre-exposure prophylaxis (PrEP). Yet, very little research has been conducted to understand the perspectives and practices of HIV-serodifferent couples regarding HIV prevention strategies in the U.S. To help fill this gap, we conducted a mixed methods study with 27 mostly Black/African American and Latinx HIV-serodifferent heterosexual couples residing in New York City to explore their knowledge, attitudes, practices, and perspectives regarding combination HIV prevention, including condoms, PrEP and viral control. All couples expressed the desire to maintain viral suppression in the HIV-positive partner, which was not always achieved. There was considerable heterogeneity in the use of HIV prevention methods by couples; and several patterns emerged that were largely driven by gender and relationship dynamics. Female partners, in particular, expressed high levels of anxiety around transmission of HIV and thus desired multiple methods of protection. Healthcare providers should consider couples’ psychosocial well-being, relationship quality, and other motivational factors when helping to tailor HIV preventative care for mixed-status couples.

## Introduction

With the advent of new strategies to prevent HIV transmission among serodiscordant couples, gaining an understanding of what factors influence and motivate serodiscordant couples to take up strategies to prevent HIV is important [1].

Heterosexual contact remains the most prevalent route of HIV transmission in many regions of the world, most notably in the context of primary or intimate relationships [2–10]. In the United States (U.S.), heterosexually acquired HIV accounts for nearly 1 in 4 new diagnoses [11]. Black/African Americans continue to be disproportionally represented among new HIV cases attributed to heterosexual transmission: they account for 61% of such cases despite making up only 13% of the U.S. population [11]. As in the global pandemic, a substantial proportion of heterosexually acquired HIV infections in the U.S. occur between partners in primary relationships characterized by mixed HIV status [7, 12, 13]. There are an estimated 140,000 such HIV-serodifferent heterosexual couples living in the U.S. [14]; and evidence suggests that uninfected persons in long-term sexual partnerships with HIV-positive individuals are at heightened risk for infection, even compared with other high-risk groups [2, 5, 8, 15–21].

Studies published within the last decade indicate that condom use varies widely among HIV-serodifferent heterosexual couples in the U.S., with 34% to 77% of couples reporting inconsistent or no condom use [22–25]. Moreover, between 45% and 60% of HIV-positive partners in serodifferent relationships do not achieve sustained viral suppression [22, 26, 27]. Based on these and other epidemiological data, the World Health Organization [28] and the U.S. Centers for Disease Control and Prevention [29] have issued guidelines prioritizing HIV-serodifferent couples for primary HIV prevention, including treatment-as-prevention (TasP) and pre-exposure prophylaxis (PrEP). Several surveys also reveal that clinical providers view HIV-serodifferent couples as a priority group for PrEP provision [30–37], although uptake of PrEP in this group has been slow [37, 38].

Despite this consensus, very little research has been conducted on HIV prevention and health promotion among HIV-serodifferent couples in Western countries, including the U.S. [12, 39–41]. To help fill this gap, we explored couples’ knowledge, attitudes, practices, and perspectives regarding HIV prevention approaches involving condom use, viral suppression in the HIV-positive partner, and PrEP use by the HIV-negative partner among 27 HIV-serodifferent couples residing in New York City.

### Theoretical perspective

The present study was guided by a conceptual framework that developed out of the need to integrate social and interpersonal dynamics with existing cognitive theories of health behavior change. Beginning in the 1990s, researchers like Mindy Fullilove [42], Hortensia Amaro [43, 44], Gina Wingood [45, 46], and others [47–51] began questioning the validity of theories of sexual health behavior that were centered on cognitive risk-reward models at the individual level. Largely motivated by the need to better understand women’s sexual health behavior [52–54], particularly in the context of intimate relationships, these authors initiated a shift toward theories with greater emphasis on social and dyadic interaction and interdependence and the key role of gender dynamics influencing sexual behavior and health.

Working within this tradition, we adopted the model proposed by Gorbach and Holmes [55] as our central conceptual framework, which emphasizes social, emotional, physical and power relationship dynamics as imperative for understanding couples’ sexual health behavior, while acknowledging the role played by individual characteristics and social context. We further integrated concepts from two models along the lineage established by Gorbach and Holmes. First, from the interdependence and communal coping model of Lewis and colleagues [56], we examined the degree to which couples are emotionally interdependent and engage in cooperative problem solving (“communal coping”) in relation to HIV prevention strategies. Second, from the dyadic framework of Karney and colleagues [57], we examined the interplay between mutual partner influence and individual attitudes, beliefs and intersectional roles, and how these dynamics might affect couples’ sexual health behavior.

## Methods

### Design, sample, recruitment and data collection

A convergent mixed-method study design [58] consisting of quantitative surveys and qualitative semi-structured interviews was performed involving a single sample (N = 54 individuals; 27 serodifferent couples) in order to identify patterns and contextualize research findings for optimal interpretation [59]. We enrolled 27 mostly Black/African American and Latinx mutually disclosed heterosexual HIV-serodifferent couples residing in New York City. Each couple’s mixed HIV status was confirmed by rapid antibody test at screening. To be eligible, HIV-serodifferent couples had to report being in a primary, sexually active heterosexual relationship for at least three months. “Sexually active” was defined as having penetrative penile-vaginal or penile-anal intercourse with a primary partner in the previous 3 months. Both members of the couple had to be at least 18 years of age, speak fluent English or Spanish, and provide written informed consent to participate in the study. The HIV-negative partner either had to be taking PrEP or have received a PrEP consultation with a health care provider and be deemed eligible for PrEP provision based on New York State or CDC guidelines at the time of the interview.

Couples were recruited between May 2015 and February 2016 from 9 clinical sites in New York City, including HIV and infectious disease specialty clinics as well as primary care clinics; the sites varied in size and type from large medical centers to smaller community clinics and were located in low income neighborhoods in upper Manhattan, Brooklyn, Queens, and the Bronx. Eligible couples visited our research field office located in mid-town Manhattan for enrollment and data collection. At the field office, members of the couple were separated into private offices for enrollment and data collection (for a description of methods see [60]). Once enrolled, each member was administered a quantitative survey and a qualitative interview. Separate interviews with members of a couple have been shown to result in less bias and permit a better understanding of couple concurrence as well as disagreement of perspectives [26, 61]. Despite the availability of Spanish language interviews, all participants preferred to be interviewed in English. Data collection activities lasted about 1.5 hours, on average. Saturation was determined by iterative review of transcripts to a point of minimal new information capture.

### Qualitative interview guide

Following a descriptive narrative approach, semi-structured qualitative interviews (all conducted by JS) were directed by a set of interview guides tailored to participant characteristics (HIV-negative partner on PrEP; HIV-negative partner not on PrEP; HIV-positive partner). Topics included couple relationship history and dynamics; attitudes and decision-making regarding HIV prevention strategies, including condoms, viral suppression, and PrEP; experiences with the healthcare system related to antiretroviral therapy (ART) or PrEP; HIV testing and psychosocial factors related to perceived HIV transmission risk; and mental health and substance use.

### Quantitative survey measures

Quantitative surveys were self-administered by audio computer-assisted self-interview (ACASI) with a touch screen tablet and headphones using QDS version 2.6 software (NOVA Research Inc.). Survey items covered topics on demographics; physical and mental health; relationship dynamics; health services utilization and experiences; HIV prevention methods, including PrEP, treatment-as-prevention, and condom use; and psychosocial and interpersonal factors related to HIV prevention. Measures employed in the current analysis included the following:

Viral suppression (dyadic). Although viral suppression pertains to the status of the HIV-positive partner of each couple, it was treated as a property of the couple and thus a dyadic-level variable. Two measures were used to quantify viral suppression: (1) self-reported undetectable viral load at last test; and (2) self-reported continuous undetectable viral load status over the past 3 years.

Condom use (dyadic): We examined self-reported condom use by the couple as a 3-level categorical variable: never, sometimes, or always. “Never” was defined as never using a condom during penile-vaginal and penile-anal acts of intercourse; “Sometimes” was defined as sometimes using a condom during sex; and “Always” was defined as consistently using a condom for every penile-vaginal and penile-anal act of intercourse. For some analyses, we dichotomized these categories (i.e., consistent condom use versus not consistent condom use).

Condom use attitude (individual). Attitude towards condoms was measured by taking the mean score on 8 items composing the Sexual Experience subscale of the Condom Barriers Scale [62, 63]. Sample questions include: “I feel closer to my partner without a condom” and “Condoms interrupt the mood,” with a 5-point Likert-type response set ranging from Strongly Agree = 1 to Strongly Disagree = 5. Higher scores indicate a more positive attitude towards condoms. Cronbach’s alpha (N = 54) was 0.87.

PrEP use (dyadic). Although PrEP use or non-use pertains to the HIV-negative partner of each couple, it was treated as a dyadic-level variable. Self-reported PrEP use was confirmed by study staff observation of a current PrEP prescription or pill bottle label.

Anxiety related to dyadic HIV-transmission (individual). Perceived “level of anxiety about the HIV-negative partner becoming infected” (i.e., within-couple HIV transmission) was measured using an ordinal scale with 3 categories: low = 1, moderate = 2, and high = 3.

HIV status (individual). The HIV-serodifferent status of couples was confirmed using the OraQuick Rapid Antibody Test Advance HIV-1/2 kit (OraSure Technologies, Inc.). A dyadic-level variable was also created indicating whether each couple was male-positive or female-positive.

Demographics (individual/dyadic). Demographic and other data were collected to describe the sample, including sex, gender, age, race, ethnicity, education, employment, income, HIV history, drug use, marital status, reproductive intentions, and sexual behavior.

### Data analysis

Qualitative data analysis proceeded as a series of sequential steps. First, all digital audio recordings were transcribed verbatim, checked for accuracy, and loaded into a cloud-based qualitative research software system (Dedoose, ver. 8.3.4; www.dedoose.com). An initial coding tree was developed deductively based on our research questions and interview topics. The coding tree was then expanded inductively and refined by adding, merging and renaming codes. This was accomplished by triple coding the first set of transcripts and discussing all differences in coding among the three coders (authors JS, NL, AB) until a consensus was achieved. This method was employed until coding discrepancies among coders were minimal (the first 12 transcripts). The remaining 15 transcripts were then coded by one of the three researchers, and these transcripts were periodically checked for accuracy. Once coding was complete, we applied a framework approach using thematic analysis to compare and contrast themes related to couples’ combination HIV prevention strategies involving condoms, viral suppression, and PrEP according to several key dimensions (sex and HIV status of the partner; relationship dynamics).

Given our focus on couples, transcripts from both partners were analyzed so that factors associated with being coupled (e.g. influence of partner, relationship history, quality of relationship) as well as other contextually relevant information (e.g. relationship with medical providers/clinics, experience of HIV-related stigma, active drug use) were also identified and analyzed (compared and contrasted).

Quantitative descriptive and inferential analyses were performed on survey data using SAS (ver. 9.4). Standard data cleaning methods were employed to assess and remedy violations of general linear model assumptions. The analysis dataset contained no missing data. Given the small samples (N = 54 for individual-level analysis; N = 27 for couple-level analysis), we had limited power to detect small or medium effect sizes. For most results, we present the point estimates of effect size, 95% confidence intervals, and p-values; however, consistent with recent guidelines of the American Statistical Association, we avoid the use of p-value cutoffs and wording related to the concept of “statistical significance” [64]. For inferential analysis, when the unit of analysis was at the individual-level (N = 54; e.g. condom use attitudes) generalized estimating equation (GEE) analysis was performed to account for clustering of individuals within dyads, generating adjusted unstandardized regression coefficients (B). When the unit of analysis was at the dyadic-level (N = 27; e.g. consistent condom use), relative risk (RR), Chi-square, Fisher’s Exact test, or mean difference t-tests were applied depending on the type of variables in the model. Results from quantitative analyses are crude (unadjusted for covariates) unless otherwise indicated.

Mixed-methods integration was performed by *connecting* and *merging* qualitative and quantitative data subsequent to parallel data collection [65]. A bi-directional iterative approach was used as part of the integrative analysis in which emergent themes from the qualitative analysis guided subsequent quantitative analysis and findings from quantitative analysis prompted exploration of qualitative concepts. The mixed-methods results are presented through a *weaving narrative* interpretation, *meta-inferences*, and *joint display* [65]. Meta-inferences involved determining the “fit” of data integration as either confirmation, expansion, or discordance. *Confirmation* occurs when both qualitative and quantitative analyses yield corroborating results; *expansion* is marked by the two types of data illuminating different or complementary aspects of the phenomenon; and *discordance* occurs when qualitative and quantitative findings are inconsistent [65].

### Ethics approval and consent to participate

The study was approved by the University of Rochester Institutional Review Board (STUDY568) in compliance with all regulations and policies regarding ethical conduct of research. All participants provided signed written informed consent to participate in the study prior to data collection.

## Results

Characteristics of the study sample are presented in Table 1. Each participant self-reported binary and congruent sex at birth and gender identity. Of the 27 couples interviewed, 16 (59.3%) had an HIV-positive female partner and 11 (40.7%) were male HIV-positive couples; 15 (55.6%) were married either legally or through common law; none were pregnant but 3 (11.1%) were trying to conceive. Examining the pooled sample (N = 54), mean age was 49.4 years (SD = 9.4); 70.4% were Black/African American, 22.2% were Hispanic/Latinx, 5.6% were White, and 1.9% were mixed race. Average annual income was $11,784 (SD = $1,061); 63% completed high school and 7.4% graduated college; 16% were employed full- or part-time; 35.2% were unemployed; 38.9% were unable to work due to disability; and 7.4% were retired. The mean duration of couples’ relationships was 6.3 years (SD = 4.9). Among HIV-positive partners, the mean number of years since diagnosis was 20.5 (SD = 7.88). Nearly one-quarter (22.2%) reported having the Hepatitis C virus; and one-third (33.3%) reported using illicit drugs (injected or non-injected) in the prior 3 months. Only two couples reported injecting drugs together.

**Table 1:** Sample characteristics (N = 54).

	Female (n = 27)	Male (n = 27)	Dyad (n = 27)
	Mean (Range)	Mean (Range)	Mean (Range)
Age (yrs)	48.5 (26–64)	50.2 (31–67)	
Annual income (USD)	$13,116 (600–73,296)	$9,744 (0–42,000)	
Lifetime number of HIV tests (HIV-negative partner only)	10.0 (1–30) ^a^	22.9 (2–90) ^b^	
Years since HIV diagnosis (HIV-positive partner only)	21.1 (9–37) ^b^	19.6 (5–28) ^a^	
Duration of relationship (years)			6.3 (0.25–21.25)
	n (%)	n (%)	n (%)
Race			
Black / African American	18 (66.7)	20 (74.1)	
White / Mixed	2 (7.4)	2 (7.4)	
Hispanic/Latinx	7 (25.9)	5 (18.5)	
Completed high school	15 (55.6)	19 (70.4)	
Employed (full or part-time)	4 (14.8)	5 (18.5)	
Hepatitis C diagnosis (ever)	6 (22.2)	6 (22.2)	
Non-injection drug use (prior 3 months)	7 (26.0)	8 (29.6)	
Injection drug use (prior 3 months)	1 (3.7)	4 (14.8)	
Marital status			
Married, legal			9 (33.3)
Married, common law			6 (22.2)
Not married			12 (44.5)
Trying to conceive			3 (11.1)
Female HIV-positive couple			16 (59.3)
HIV-negative partner on PrEP	9/11 (81.8)	7/16 (43.8)	16 (59.3)
Frequency of vaginal-penile sex per week ^c^			2.22 (1–6)
Engaged in anal-penile sex in prior week			6 (22.2)

a. n = 11

b. n = 16

c. 5% trimmed mean and range

### Couple’s HIV prevention strategies

**Fig 1:**
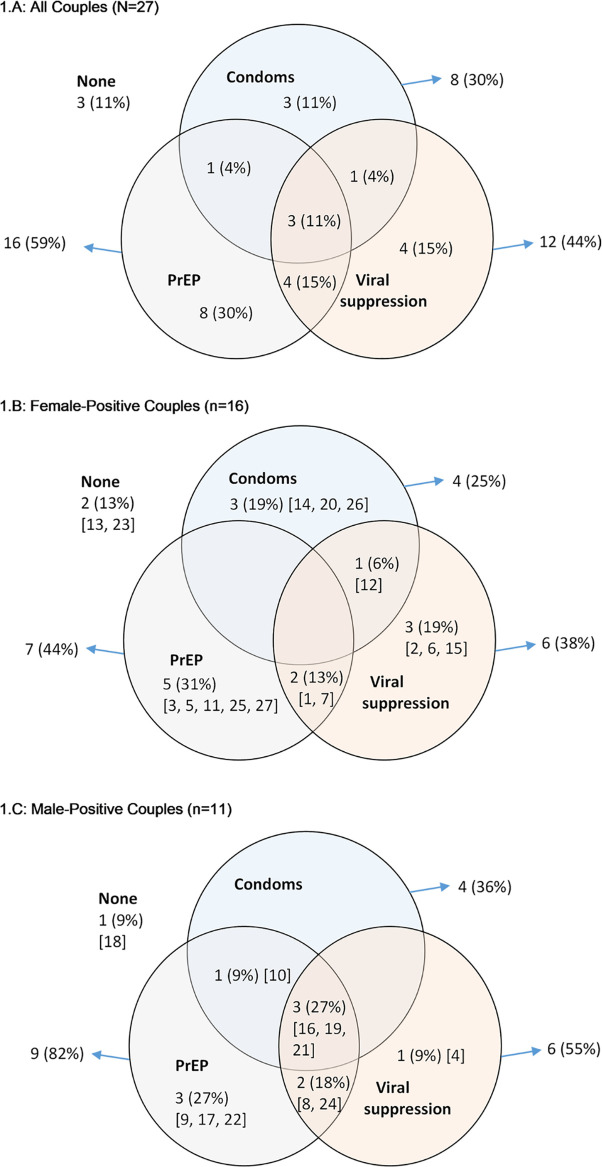
Venn diagram of combination HIV prevention strategies used by HIV-serodifferent couples. Condoms = Consistent condom use for vaginal and anal intercourse; Viral suppression = sustained viral suppression; PrEP = HIV-negative partner currently prescribed PrEP; None = None of the three types of prevention methods used; Couple ID numbers appear in brackets.

Consistent with our eligibility criteria, all couples were sexually active and none mentioned abstinence as a strategy to prevent HIV transmission within the partnership. Apart from several ad hoc comments about withdrawal (i.e., coitus interruptus), genital washing after intercourse, or avoidance of anal sex as methods for HIV prevention, nearly all of the strategies discussed by couples for preventing dyadic HIV transmission centered on some combination of viral suppression, condom use, and PrEP (see Fig 1): 8 (29.6%) couples reportedly used condoms consistently; 12 (44.4%) HIV-positive partners reported sustained undetectable viral load; and 16 (59.3%) had an HIV-negative partner who was taking PrEP. The Venn diagram (Fig 1A) shows the combination of these three HIV prevention strategies among couples in the sample. Three couples did not use any of the three methods at the time of the interview, whereas 16 couples used only a single method (4 used viral suppression; 3 used condoms; 9 used PrEP) and 9 used more than one method (3 used PrEP/viral suppression; 1 used PrEP/condoms; 1 used condoms/viral suppression; 3 used all three methods). Fig 1B and 1C present the combination of HIV prevention methods by the sex of the HIV-positive partner. Female HIV-positive couples were more likely to rely on only a single prevention method (68.8%), whereas male HIV-positive couples were more likely to rely on combination prevention (63.6%), especially PrEP combined with some other method.

### Mixed methods results

**Fig 2:**
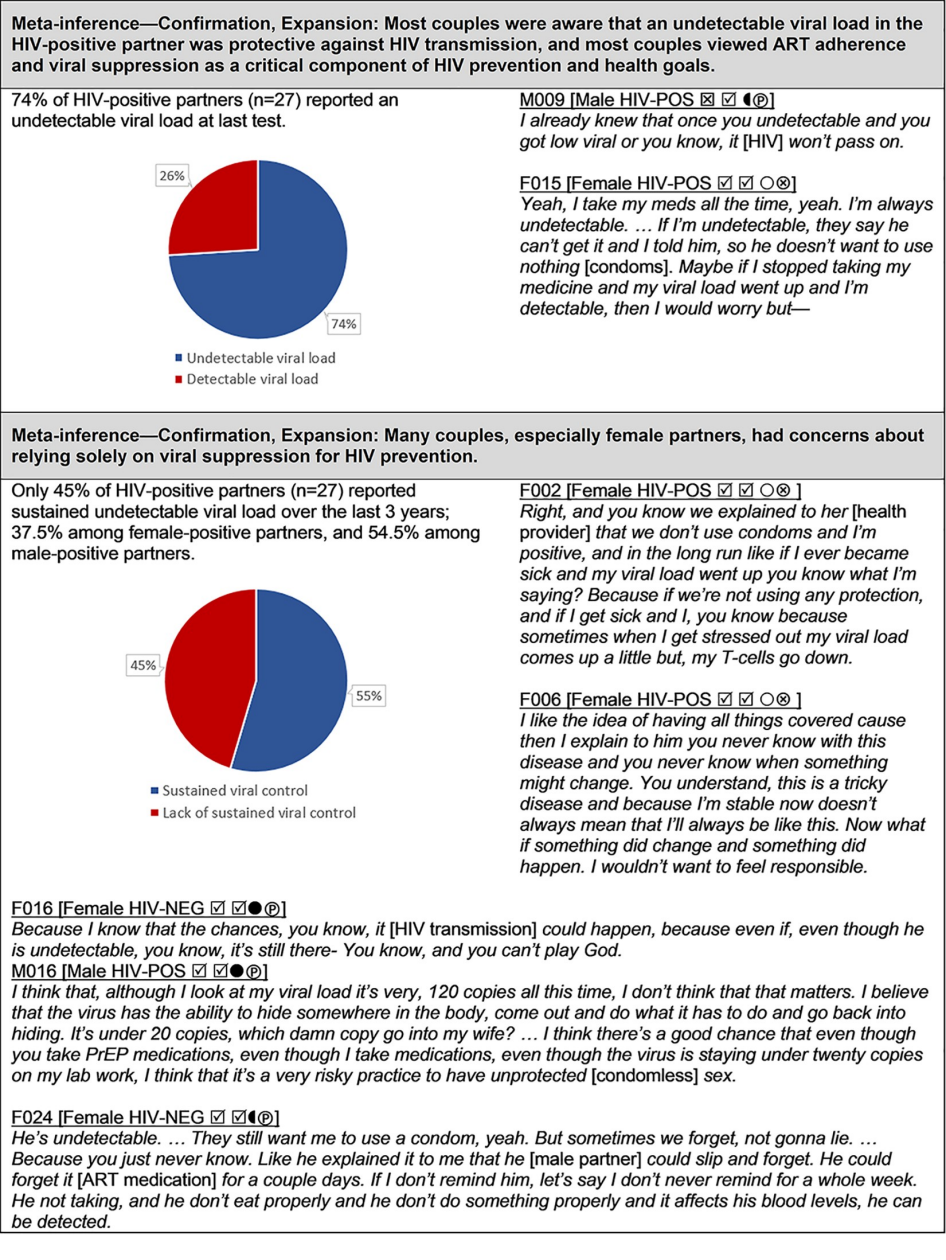
Viral suppression.

**Fig 3:**
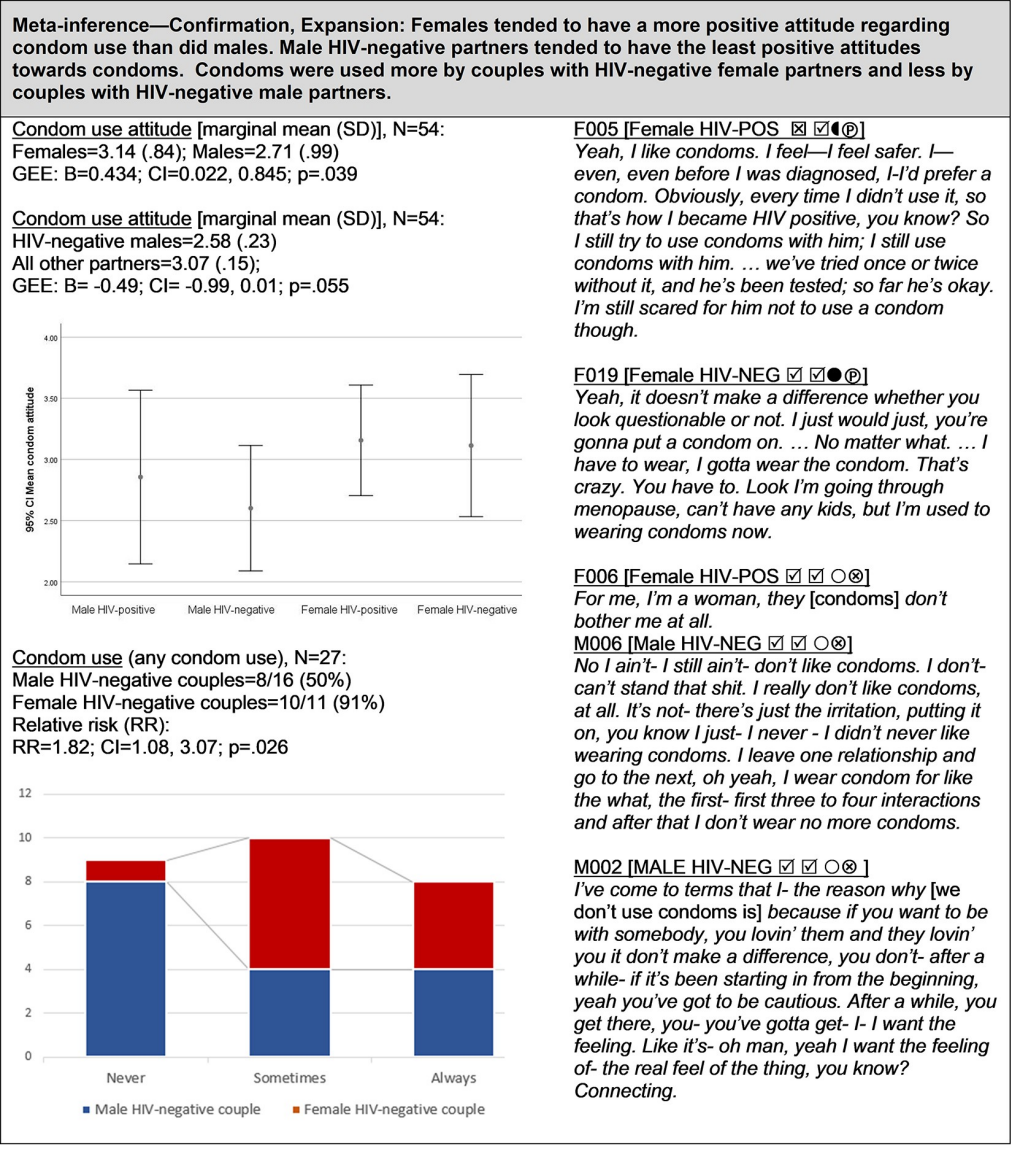
Condom use.

**Fig 4:**
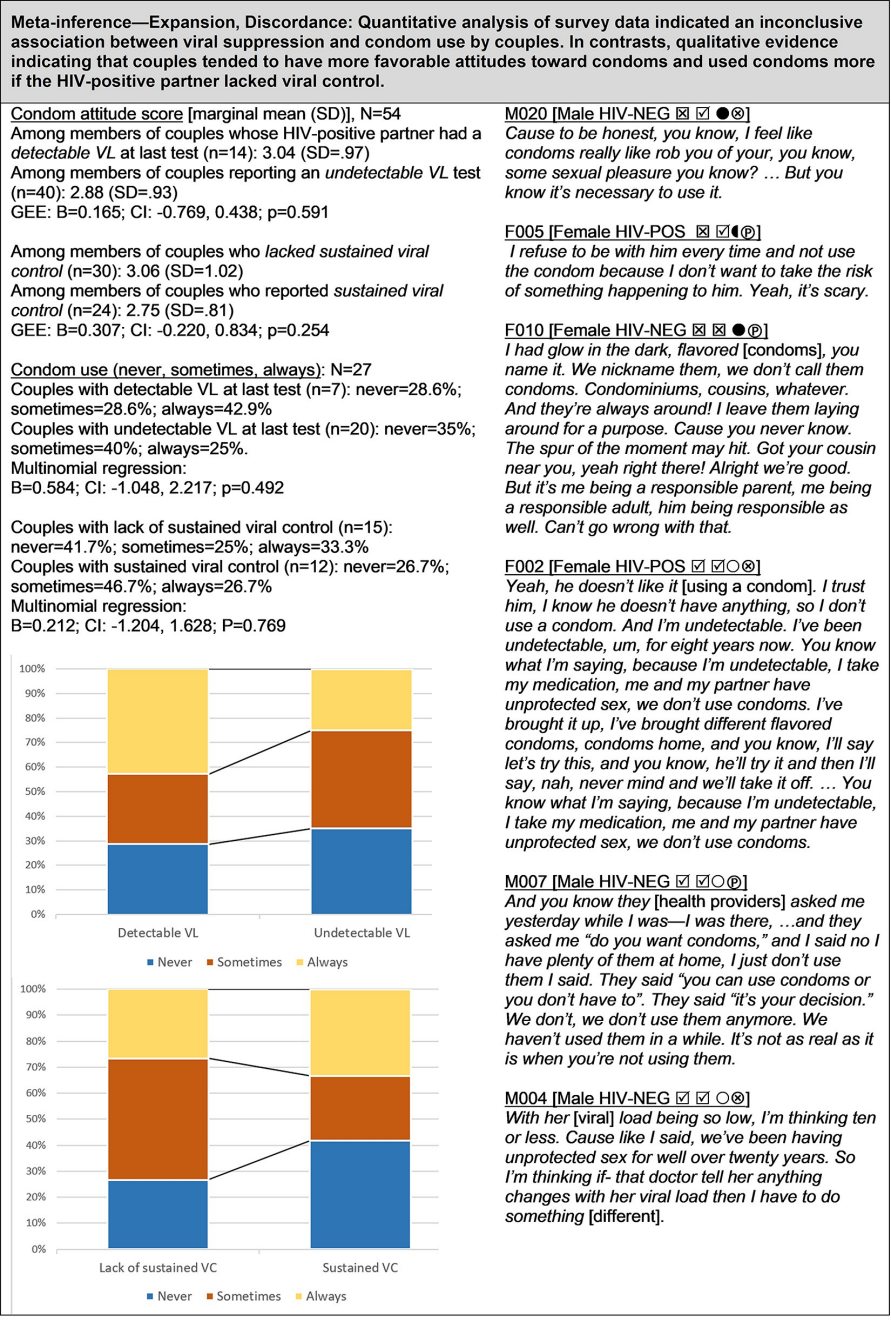
Viral suppression and condom use.

**Fig 5:**
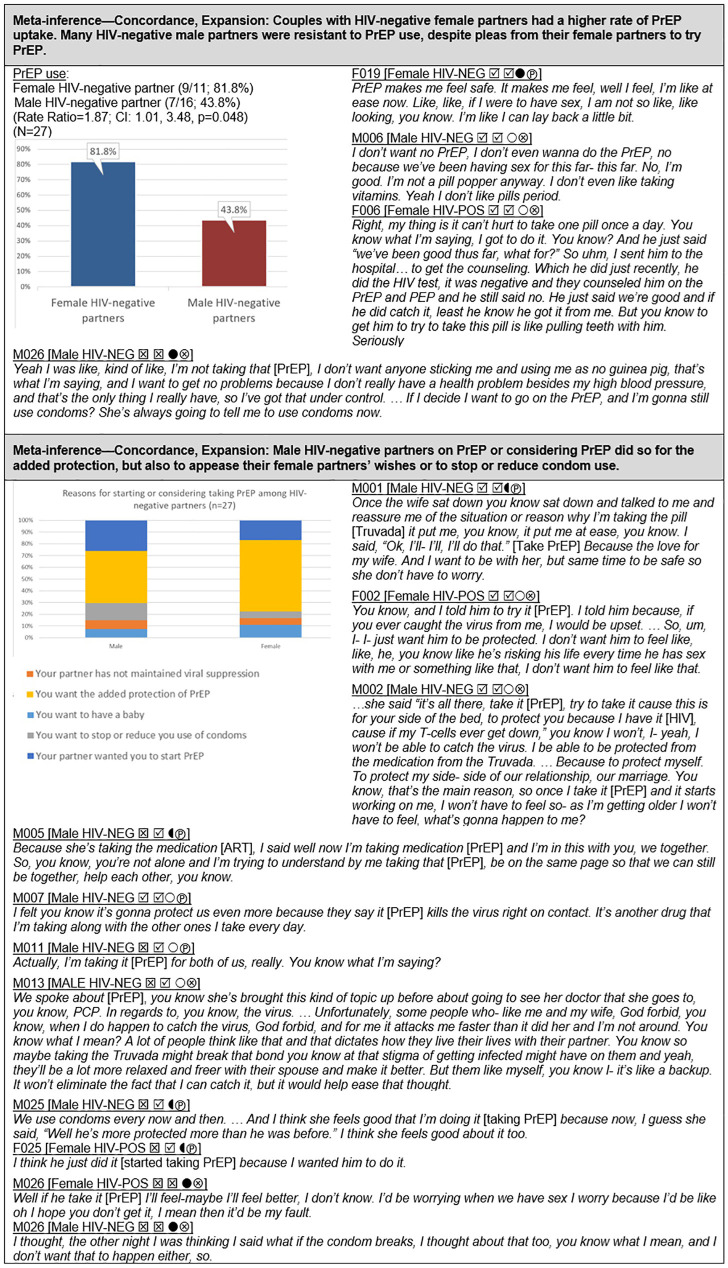
PrEP use.

**Fig 6:**
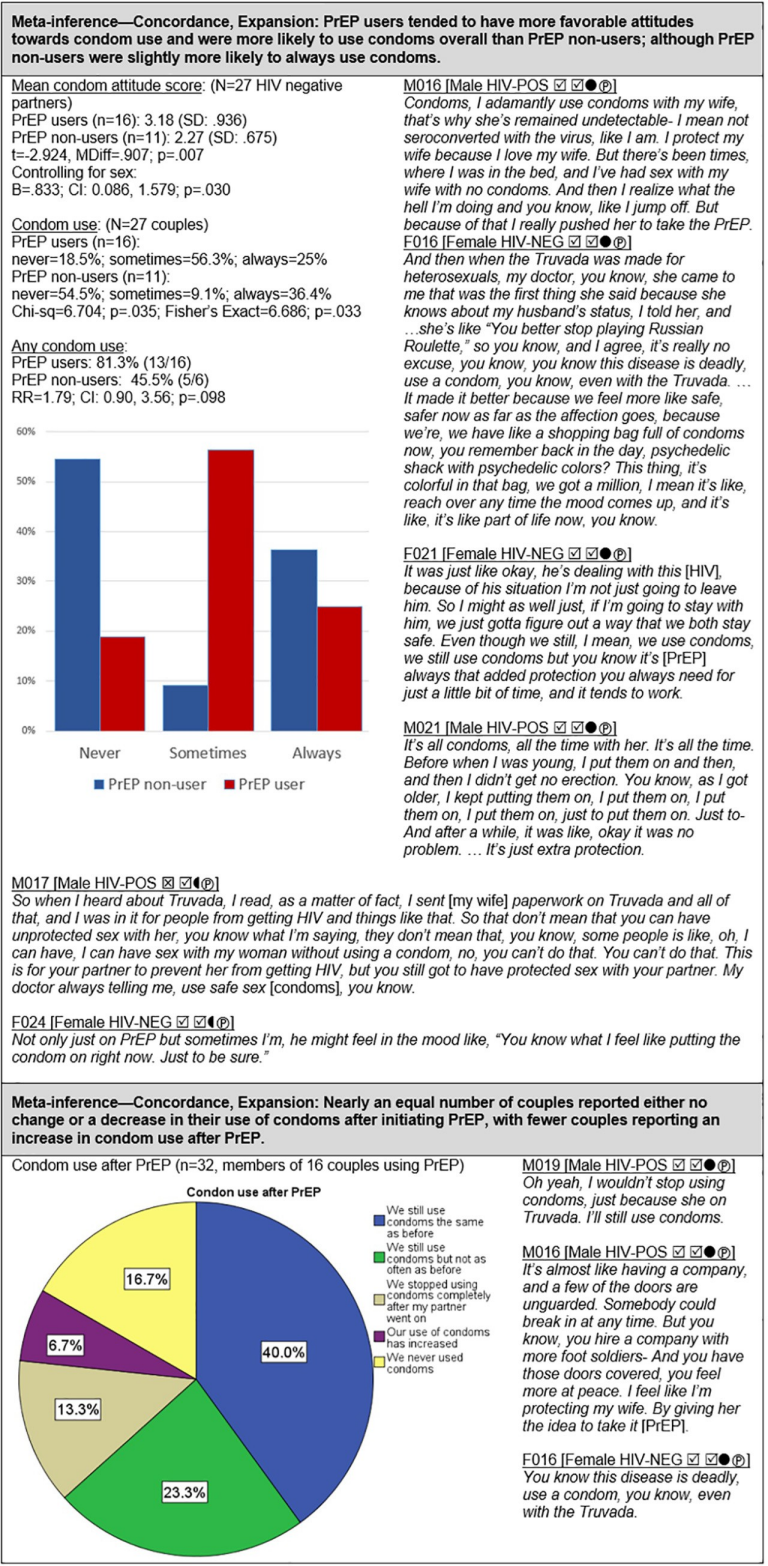
PrEP and condom use.

**Fig 7:**
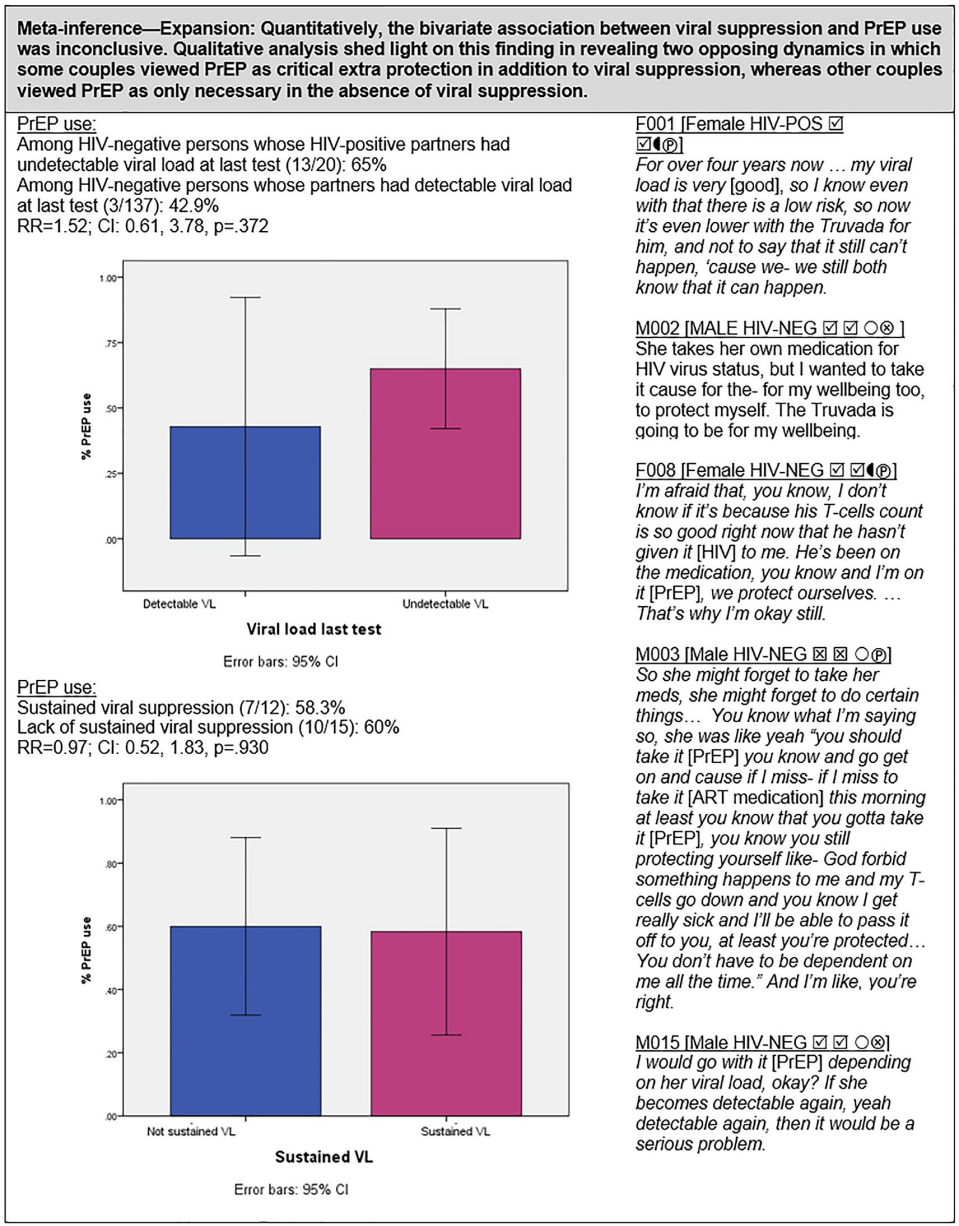
PrEP and viral suppression.

**Fig 8:**
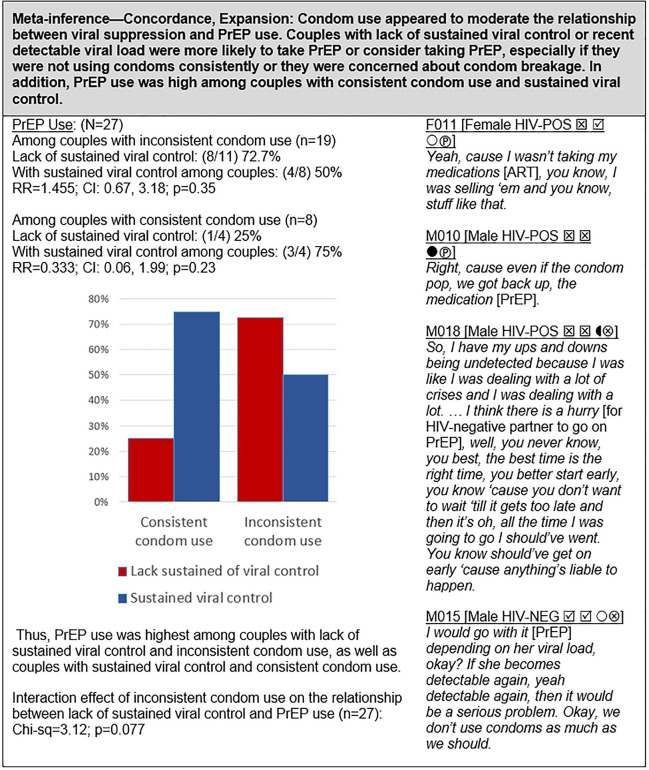
Viral suppression, condoms and PrEP.

**Fig 9:**
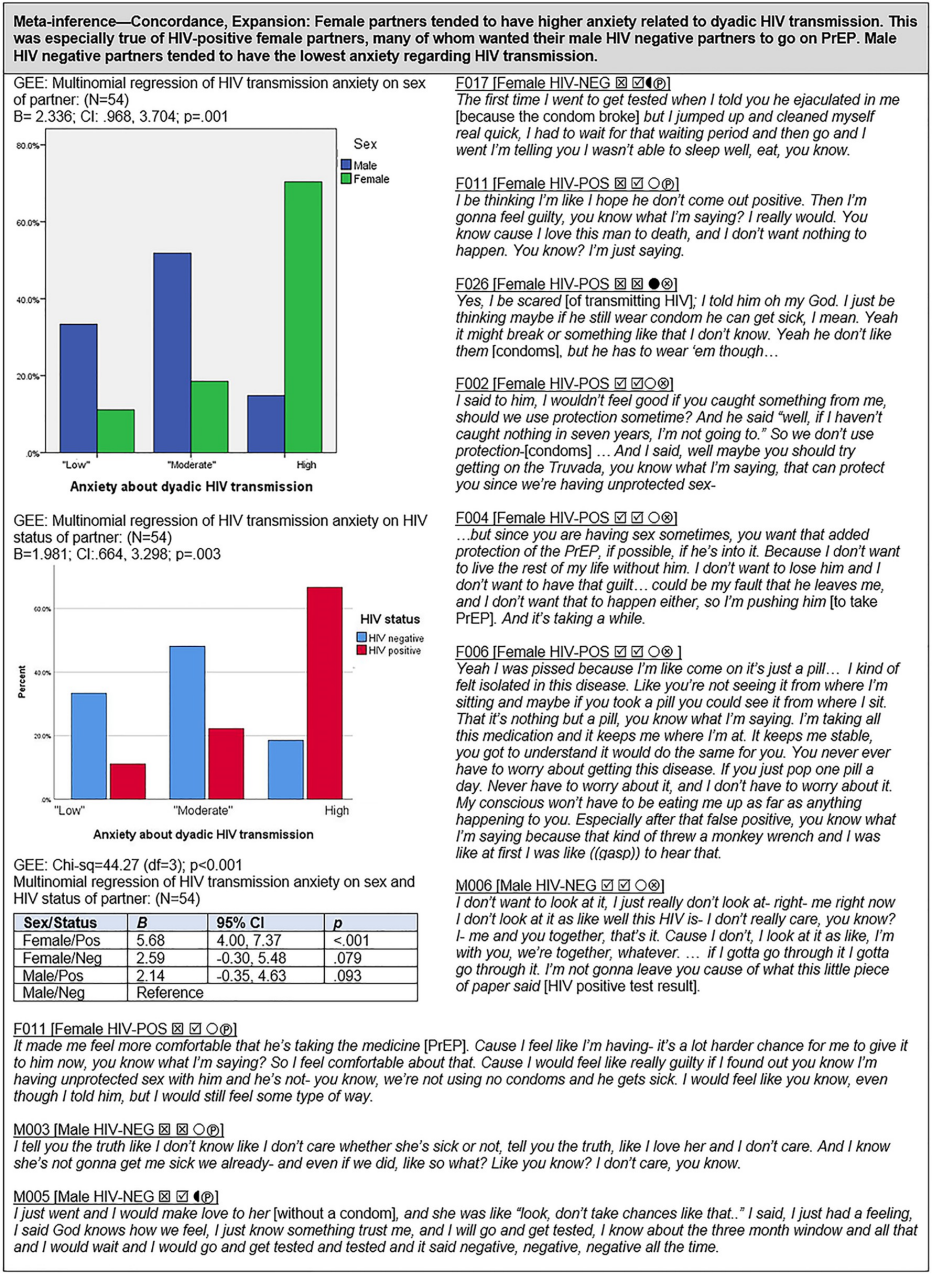
HIV transmission anxiety.

Figs 2–9 provide narrative interpretation, meta-inferences, and joint data displays of the mixed methods results. Each figure addresses an HIV prevention method, or combination of methods, and is labeled with the relevant theme, followed by meta-inference (confirmation, expansion, or discordance) and a summary interpretation. For joint displays, quantitative results (left side panel) are provided in both graphic and numeric form and qualitative results (right side panel) are comprised of selected excerpts from interviews, and are identified by participant ID (e.g., M009 indicates male partner from couple 009), HIV status (HIV-POS, HIV-NEG) and a series of four symbols representing combination HIV prevention methods of the couples: the first symbol indicates whether the HIV-positive partner reported long-term sustained viral suppression (☒ = No, ☑ = Yes); the second symbol indicates HIV suppression status at last test reported by the HIV-positive partner (☒ = Detectable, ☑ = Undetectable); the third symbol indicates condom use by the couple (● = All the time; ◖ = Some of the time; ○ = None of the time); and the fourth symbol indicate whether the HIV-negative partner is on PrEP (⊗ = No; Ⓟ = Yes).

#### Viral suppression (Fig 2)

Nearly all couples expressed a desire for the HIV-positive partner to stay adherent to their antiretroviral regimen in order to remain healthy and reduce the risk of transmitting HIV. During the time of the interviews, the concept of U = U (Undetectable = Untransmittable) had not yet been established, but most couples had either heard that maintaining a low viral load in the positive partner reduced the risk of HIV transmission or intuited this from their own experience as part of an HIV-serodifferent couple. When asked about their last viral load test, 74.1% (20/27) of HIV-positive partners reported receiving an undetectable viral load result and 25.9% (7/27) reported having a detectable viral load. When asked whether they had been able to sustain an undetectable viral load status consistently for the last 3 years, only 44.4% (12/27) responded in the affirmative. Sustained undetectable viral load was reported by only 37.5% (6/16) of female HIV-positive partners, whereas 54.5% (6/11) of male HIV-positive partners reported sustained viral suppression. The low prevalence of sustained viral suppression among many HIV-positive partners was reflected in concerns about relying solely on viral suppression as a means of protection among some couples. In contrast, a minority of participants expressed high confidence that viral control alone was sufficient to prevent HIV transmission.

#### Condom use (Fig 3)

Of the 27 HIV-serodifferent couples interviewed, 9 (33.3%) reported never using condoms, 10 (37%) reported using condoms some of the time, and 8 (29.6%) stated that they used condoms all of the time. All participants completed the Condom Sexual Experience Scale, for which higher scores indicated a more positive attitude towards condom use with their primary partner. Overall, female partners tended to view condoms more favorably than males (B = .43; CI: .02, .85); and couples with HIV-negative female partners were nearly twice as likely to report any condom use compared to couples with male-negative partners (RR = 1.82; CI: 1.08, 3.07). In contrast, couples with HIV-negative male partners were over six times more likely to never use condoms (RR = 6.19, CI: .91, 42.12). When asked to rate their desire to use condoms with their primary partner (on a scale of 1–5, with 5 as the highest desire), both male and female HIV-positive partners reported high scores (median = 4); female HIV-negative partners also expressed high desire to use condoms (median = 4); whereas, male HIV-negative partners reported relatively low desire to use condoms (median = 2). Thus, HIV-positive partners of both sexes tended to want to use condoms to protect their negative partner, but among HIV-negative partners, females expressed a greater desire to use condoms than did males. These sex-based preferences of the HIV-negative partner translated into more condom use among female-negative couples than male-positive couples.

#### Viral suppression and condom use (Fig 4)

The relationship between viral suppression and condom use among HIV-serodifferent couples is complex and potentially discordant by data type: quantitative analysis returned inconclusive results with confidence intervals containing positive, negative, and null effects, whereas qualitative analysis revealed that participants expressed more favorable attitudes towards condoms and condom use if the HIV-positive partner had a detectable viral load at their last test or was not able to sustain viral suppression. Conversely, couples who were confident about and had demonstrated sustained viral suppression in the HIV-positive partner tended to be less anxious about the need for condoms compared to those with a history of detectable viral load results. Descriptive data (point estimates) from the survey supported this observation: 43% of couples with a detectable VL at last test reported always using condoms, compared to only 25% consistent condom use by couples with an undetectable VL at last test. However, the *pattern* of condom use in relation to sustained viral control was more complex. Although a higher proportion of couples with sustained viral control reported never using condoms (42%) compared to couples with lack of sustained viral control (27%), a counterintuitive pattern emerged revealing that a greater proportion of couples with sustained viral control reported always using a condom (33%) compared to couples with lack of sustained viral control (27%).

#### PrEP use (Fig 5)

Among the 27 HIV-serodifferent couples who participated in the study, 16 (59.3%) of the HIV-negative partners were being prescribed PrEP and 11 (41.7%) were not. The prevalence of PrEP use varied according to the sex of the HIV-negative partner. Nine of the 11 female-negative partners were using PrEP (81.8%), whereas only 7 of 16 male-negative partners were on PrEP (43.8%) (RR = 1.87; CI: 1.01, 3.48). Qualitative evidence showed that HIV-negative male partners were less concerned about or perceived less risk of transmission compared to their female-negative counterparts; some HIV-negative male partners were against taking PrEP due to medication aversion or distrust of the medical establishment, whereas others conceded to taking PrEP to reduce condom use, to alleviate their partner’s anxiety, to strengthen the relationship by demonstrating shared responsibility for HIV prevention, and for the added protection against HIV.

#### PrEP and condom use (Fig 6)

It is of interest to note that among HIV-negative participants, PrEP users had more favorable attitudes towards condoms than did PrEP non-users, even after controlling for sex (B = .833; CI: 0.086, 1.579; p = .030). This translated into higher use of condoms (any condom use) among couples using PrEP compared to PrEP non-users (RR = 1.79; CI: .90, 3.56). Couples who reported never using condoms were predominately male-negative (89%) and had a low prevalence of PrEP use (33%). Thus, compared to female-negative couples, male-negative couples tended to report lower PrEP use despite also reporting lower condom use and having female-positive partners with lower sustained viral control.

Members of couples using PrEP were asked whether and how initiating PrEP changed their use of condoms; a plurality (40%) reported that PrEP had not affected their condom use, whereas nearly as many (36.6%) indicated that they had either decreased or stopped using condoms after initiating PrEP. In contrast, only a few (6.7%) reported an increase in condom use after starting PrEP. In addition, all participants were asked to indicate how strongly they agreed or disagreed with the following statement: “If an HIV-negative person is taking Truvada/PrEP every day, then there is no need for them to use condoms when having sex with an HIV-positive person” (strongly agree = 5 to strongly disagree = 1). A majority of participants (33/54; 61.1%) disagreed with this statement: 65.6% (21/32) of PrEP users disagreed, whereas 54.5% (12/22) of PrEP non-users disagreed, and these attitudes were reflected in substantially higher use of condoms among PrEP users (81.3%) compared to PrEP non-users (45.5%).

#### PrEP and viral suppression (Fig 7)

We examined whether having an undetectable viral load at last test or maintaining viral suppression was associated with PrEP use. Among couples with an undetectable viral load at last test, 65% (13/20) were taking PrEP compared to 42.9% (3/7) among couples with a detectable viral load at last test (RR = 1.52; CI: 0.61, 3.78; p = 0.37). PrEP use was nearly identical between couples with sustained viral control (58.3%; 7/12) compared to couples with lack of sustained viral control (60%; 9/15) (RR = 0.97; CI: 0.52, 1.83). Thus, quantitatively, the potential effect of viral control on PrEP use was inconclusive. Qualitative data may help shed light on the lack of quantitative findings in revealing different motivations for PrEP use: some couples viewed PrEP as essential extra protection in addition to viral suppression, whereas other couples regarded PrEP as necessary only in the absence of viral suppression.

#### Viral suppression, condoms and PrEP (Fig 8)

Quantitative and qualitative analyses revealed a complex relationship among viral suppression, condom use and PrEP, in which PrEP use was highest among couples with consistent condom use and sustained viral control, but nearly as high among couples with inconsistent condom use and lack of sustained viral suppression. In contrast, PrEP use was lowest among couples with consistent condom use but who lacked viral suppression, and PrEP use was intermediate among couples with inconsistent condom use and sustained viral control.

#### Anxiety regarding HIV transmission (Fig 9)

We explored participant’s perceived anxiety around HIV transmission within the dyad, with the view that this would play a significant role in shaping couple’s HIV prevention strategies. Our analysis revealed several patterns related to the sex and HIV status of partners. In general, female partners tended to express higher anxiety related to dyadic HIV transmission compared to male partners (B = 2.336; CI: .968, 3.704; p < .001); similarly, HIV-positive partners also tended to report higher HIV transmission anxiety compared to HIV-negative partners (B = 1.981; CI:.664, 3.298; p = .003). This yielded a clear pattern in which female HIV-positive partners reported the highest levels of HIV transmission anxiety, followed by female HIV-negative partners and male HIV-positive partners. Male HIV-negative partners reported the lowest levels of dyadic HIV transmission anxiety. Of the 16 HIV-negative male partners, most reported low to moderate anxiety with regard to acquiring HIV from their female partner, regardless of whether or not they were on PrEP.

Male partners expressed several reasons for these attitudes, including a long sexual history with their partner without becoming infected, denial or avoidance of confronting the couples’ HIV-serodifferent status, the female partner’s undetectable viral load, and a more accepting attitude towards becoming HIV infected. Male partners taking PrEP did so as a “backup” to either viral suppression or condoms but also to relieve their female partner’s anxiety. Several males not taking PrEP expressed these same reasons for considering PrEP use in the future. The HIV-positive female partners expressed near unanimity in reporting high anxiety around potentially transmitting HIV to their male partners. Females whose partners were on PrEP expressed much relief in having the extra protection. Among HIV-positive females whose partners were not taking PrEP, all but one wanted their partner to initiate PrEP use. These views were consistent regardless of whether or not couples were using condoms consistently or the extent of viral suppression.

In contrast, most HIV-negative female partners expressed high to moderate perceived anxiety regarding potential HIV acquisition from their male HIV-positive partners. Some expressed the view that their partner’s viral load could increase, putting them at risk, while other’s worried about inconsistent condom use or condom breakage. These female partners reported that being on PrEP gave them a feeling of added protection and control, and substantially lowered their anxiety, allowing them to relax, and their HIV-positive male partners of these women reported that PrEP use by their partners greatly reduced their anxiety about potentially transmitting the virus. They also expressed relief that their HIV-negative female partners were sharing in the responsibility of staying safe and protected.

## Discussion

This study explored HIV-serodifferent heterosexual couples’ knowledge, attitudes, perspectives and practices regarding HIV prevention strategies. We found substantial heterogeneity in perspectives and use of combination HIV prevention methods among couples. Yet, several patterns also emerged, largely driven by sex/gender and relationship dynamics. Female partners tended to have higher anxiety regarding HIV transmission, and this intersected with HIV status to generate several distinct patterns. Female partners living with HIV were the most apprehensive about relaying solely on viral suppression for prevention and many urged their partners to use condoms or initiate PrEP, which was often met with reluctance or defiance by the male partner; female HIV-negative partners were more willing to use PrEP than their male counterparts, were more engaged in supporting ART adherence in their male partners, and couples with HIV-negative female partners were more likely to use multiple forms of prevention. The following discussion places our findings in the context of our theoretical framework and prior findings in the literature on HIV prevention among heterosexual HIV-serodifferent couples, and draws parallels, contrasts, and expansions of existing theory and evidence.

### Viral suppression (Fig 2)

In addition to endorsing ART adherence as a means of promoting good health in the HIV-positive partner, nearly all couples were aware that an undetectable viral load was protective against HIV transmission (even prior to the U = U campaign). Thus, couples viewed ART adherence and viral suppression as a critical component of HIV prevention and health goals. This finding is consistent with prior evidence indicating that HIV-serodifferent couples are nearly unanimous in considering HIV treatment a high priority [66, 67].

Also consistent with previous studies [13, 66, 68, 69], we found that many couples expressed concerns about relying solely on viral supression as a means of preventing HIV transmission within the relationship. Such concerns were reinforced by the observation that more than half (15/27; 55.6%) of HIV-positive partners in our study reported not being able to maintain long-term viral suppression. Marks et al. [70] examined viral load data over time (median 3 years) among a cohort of 14,532 HIV-positive patients from six clinical sites across the U.S., and found that 54.4% had at least 1 viral load count >1500 copies/mL. Some studies suggest that being in an HIV-serodifferent relationship is associated with maintaining lower viral plasma loads [71], but the few studies that have examined viral suppression in heterosexual mixed-status couples indicate that between 45% and 60% do not achieve viral suppression [22, 26, 27], which can lead to high rates of HIV transmission [72].

Only 4 of the 27 couples (all female-positive) in our study relied exclusively on viral control to prevent dyadic HIV transmission and also reported long-term viral suppression. The HIV-negative members of these couples were acutely aware of their partners’ undetectable viral load and had gained confidence through many years of having condomless sex without transmission bolstered by repeated negative HIV tests, a finding also reported by Mahoney et al. [73]. These results underscore the need for close long-term monitoring of viral load by providers and their HIV-positive patients in the context of serodifferent couples, as well as the need to consider the psychosocial aspects of prevention strategies among HIV-affected couples. As stated by Buchbinder [74]: “In counseling patients about the risk of HIV acquisition from a sexual partner with full viral suppression, the durability of viral suppression and the consistency of medical care of the partner must be understood.”

### Condom use (Fig 3)

Prior studies have identified several factors associated with inconsistent condom use among HIV-serodifferent couples, including low education and income [26, 75], longer duration of the relationship [26], poor mental health and substance use disorders [75–77], perceived low HIV risk [78, 79], and the desire for sexual pleasure and intimacy [73]. Lack of willingness to use condoms by men [80, 81] combined with women’s limited agency to negotiate condom use in relationships often marked by gender-based power imbalances or violence has also emerged in the literature as a primary reason for low condom use in mixed status relationships [81–85].

Consistent with this body of evidence, male partners in our study expressed more negative attitudes towards condoms than did females. We also found that condom use was associated with the sex/gender of the HIV-negative partner: couples with female-negative partners tended to use condoms more than couples with male-negative partners. Consistent with this finding, studies conducted in Kenya [84] and Australia [86] have observed greater resistance to using condoms among HIV-negative male partners compared to HIV-negative female partners in serodifferent heterosexual couples; and a study involving Puerto Rican serodifferent couples reported that denial of risk by HIV-negative male partners was associated with low condom use [87]. These findings suggest that HIV-negative status within serodifferent couples may confer some degree of agency within the relationship with regard to sexual decision-making [88]. As Jones et al. have commented “the member of the couple with the greatest power may more directly influence dyadic decision-making and thereby encourage either safe or risky sex” [89]. Indeed, Kelly et al. [80] found “a shift in ethno-gender power relations towards the women within couples where the man was HIV-positive”; and other studies have reported that higher self-efficacy to discuss condom use by HIV-negative females in serodifferent heterosexual relationships was associated with higher condom use [83, 90].

In contrast, several studies have found no evidence of differences in condom use based on the sex/gender of the HIV-positive partner among mixed-status couples. In a study involving 123 HIV-serodifferent heterosexual couples in New Jersey, Skurnick et al. [77] hypothesized that the “gender of the positive partner would be associated with risk-related sexual behavior,” including condom use, but found no conclusive evidence to support their hypothesis. Similarly, in an analysis of data from 122 mixed-status couples from the California Partners Study II, Buchacz et al. [75] reported an inconclusive finding when comparing the odds of inconsistent condom use among male HIV-positive couples with those of female HIV-positive couples (OR = 1.1; 95% CI: .05, 2.2).

### Viral suppression and condom use (Fig 4)

Quantitative analysis examining the relationship between viral suppression and condom use yielded inconclusive results, with 95% confidence intervals spanning negative, null, and positive effects (Fig 4). However, analysis of qualitative data revealed that many couples tended to have more favorable attitudes toward condoms and used condoms more if the HIV-positive partner lacked viral suppression. Prior quantitative and qualitative studies that have explored the relationship between condom use and severity of HIV disease, ART adherence, or HIV biomarkers have reported mixed results. van der Straten et al. [26] examined the association between self-reported condom use and viral suppression in a sample of 104 HIV-serodifferent heterosexual couples in California and found no conclusive support for a link between viral suppression and condom use, although some discrepancies between HIV-negative and HIV-positive partners were noted. In a qualitative study of women living with HIV, Stevens et al. [81] found that condom use with HIV-negative male partners was not linked with severity of HIV symptoms. Suzan-Monti et al. [91] examined the association between sustained undetectable viral load and condom use among 656 HIV-positive men and women in serodifferent relationships in France and failed to reject the null hypothesis of no association.

In contrast, other studies have reported a link between ART and condom use among HIV serodifferent heterosexual couples. A study of 271 Canadian women living with HIV and who were undetectable at last viral load test found that those who were aware of the prevention benefits of ART were nearly twice as likely to report condomless sex with their male partner as compare to those who were not aware of the prevention benefits of ART [92]. Analysis of data from 122 HIV-serodifferent heterosexual couples enrolled in the California Partners Study II revealed that receiving ART and a higher CD4 count in the HIV-positive partner was associated with inconsistent condom use [75]. Skurnick et al. [77] followed 104 HIV-serodifferent heterosexual couples who used condoms consistently at baseline and found that less advanced stage of HIV disease in the infected partner was associated with a lapse to condomless sex after six months. Analysis of data from the Women’s Interagency HIV Study (1996–2003) revealed that among HIV-positive women in established heterosexual relationships the odds of inconsistent condom use were 22 percent higher after initiating ART compared with a pre-ART period [93], and condom use was lower among those who believed ART to be protective [94]. Analysis of longitudinal data from the Swiss HIV Cohort study reported an accelerated increase in condomless sex among HIV-positive heterosexuals in stable relationships following the “Swiss statement” declaring no risk of dyadic HIV transmission with sustained viral suppression [95]. Finally, semi-structured qualitative interviews with 12 HIV-positive partners in serodifferent heterosexual relationships found that rationalizations for condomless sex relied on lack of HIV transmission over time and the security of an undetectable viral load in the HIV-positive partner [80].

These conflicting results might be explained, in part, by a number of factors, including variations in study design, sample size, year of data collection, measures used, and study population. Findings from our qualitative interviews, and supporting evidence described above, indicate that HIV-serodifferent heterosexual couples’ use of condoms may be influenced by ART adherence and the viral load status of the HIV-positive partner.

### PrEP use (Fig 5)

In our study, female HIV-negative partners were nearly twice as likely to be taking PrEP compared with HIV-negative male partners. To-date, most studies reporting on sex-based or gender-based differences involving PrEP use among HIV-serodifferent heterosexual couples have examined willingness to use PrEP rather than actual PrEP use. In contrast to our findings, such studies have reported either little difference in willingness to take PrEP by the sex of the HIV-negative partner or a higher willingness among male HIV-negative partners. A survey of 351 HIV-negative partners in serodifferent couples conducted in three cities in China found that a similar percentage of men and women expressed willingness to use PrEP (83% and 84.9%, respectively) [96]. A survey of 181 Kenyan serodifferent couples found that a higher percentage of male HIV-negative partners were willing to use PrEP (93.8%) compared to female HIV-negative partners (86.1%) [97]. Similarly, a qualitative study of 13 HIV-negative partners in heterosexual serodifferent stable relationships in Australia also found that more men than women expressed a willingness to try PrEP [86]. This gender-based difference largely centered on men’s expectation that PrEP would allow for less condom use thereby enhancing sexual pleasure without increasing risk of HIV transmission; whereas women appeared content with the protection afforded by condoms and did not envision any reduction of condom use with PrEP uptake. Women also expressed concerns about the need for daily adherence and the effectiveness of PrEP [86].

Reluctance to use PrEP by HIV-negative male partners in our study was primarily due to perceived low HIV risk, endorsed by many years of being sexually active with their partner without HIV acquisition, and with or without condoms. In addition, several males reported that their HIV-positive female partner would continue to insist on condom use regardless of their PrEP use and, therefore, these males did not see any benefit from using PrEP. By contrast, HIV-negative females in our study perceived greater risk of HIV transmission than did their male counterparts, with several expressing concerns about lack of ART adherence by their HIV-positive male partners. Relatedly, female partners also expressed higher levels of stress and anxiety around potential transmission (see Fig 9). Females in our study may also have placed a higher premium on strengthening the relationship through shared responsibility of HIV prevention, and therefore viewed their PrEP use as a means of achieving this goal [13, 98].

Prior studies have found that HIV-negative male partners in serodifferent relationships tend to consider the management of health care, including HIV prevention, to be the responsibility of the female partner, whereas female seronegative couples tend to be more egalitarian regarding shared responsibility for HIV prevention [1, 99]. Medication aversion and medical mistrust also emerged as reasons for the lack of PrEP uptake among male partners. Few prior studies have examined sex/gender-based differences in these factors. Other explanations for the observed higher uptake of PrEP among female HIV-negative partners in our study include greater agency and motivation among females to use the first fully autonomous HIV prevention method under their control [68, 100].

### PrEP and condom use (Fig 6)

There are seemingly inconsistent findings in the literature on the impact of PrEP initiation on subsequent condom use among heterosexual HIV serodifferent couples. Some studies have found no change in condom use behavior or an increase in condom use following couples’ adoption of PrEP [101–103]. Other studies have reported that the desire to reduce or maintain low condom use is a motivating factor for initiating PrEP among some serodifferent couples [13, 86, 98, 104]. Across several studies involving HIV serodifferent heterosexual couples surveyed prior to the availability of PrEP, between 12% to 25% anticipated that they would reduce or stop using condoms if they started taking PrEP [24, 96, 105]. Among the 16 PrEP-using couples in our study, 9 (56%) reported no change in condom use, 4 (25%) reported decreased condom use, and 3 (19%) increased their use of condoms after initiating PrEP.

Further examination revealed that these results were aligned with underlying sex/gender-based dynamics. For male HIV-negative partners, intended condom use after PrEP initiation always matched actual condom use by the couple: either no change or decreased condom use. In contrast, all of the female HIV-negative partners desired either no change or increased condom use with PrEP uptake, but these desires were often ignored in favor of decreased condom use in the couple. Moreover, the 3 couples who increased their use of condoms after starting PrEP all had female HIV-negative members who reported high motivation for such an increase. Consistent with our findings, a qualitative study by Falcao et al. involving 13 HIV-negative partners in heterosexual serodifferent relationships found that female partners generally did not expect condom use behavior to change after PrEP uptake, whereas nearly all male partners anticipated a reduction in condom use with PrEP initiation [86]. This is further supported by studies in which women mostly viewed PrEP as additional protection against HIV, not as a substitute for condoms [106, 107]. Variation across studies in reported condom use after PrEP initiation by heterosexual HIV-serodifferent couples may therefore be a function, at least in part, of sex/gender composition and couple dynamics.

Sex/gender-based dynamics may also help explain our finding that PrEP users tended to have more favorable attitudes towards condom use and were more likely to use condoms than PrEP non-users. A higher proportion of HIV-negative women in our study were using PrEP compared to HIV-negative men, and, as noted, females tended to have more positive attitudes regarding condoms and greater reluctance to reduce condom use after initiating PrEP. Yet, our finding of a positive association between PrEP use and condom use held even after controlling for the sex/gender of the HIV-negative partner, indicating that some other factors might also be at play. In a study of 236 Chinese serodifferent heterosexual couples, Mijiti et al. [96] found that among those willing to take PrEP, 85% used condoms consistently in the last 6 months, whereas consistent condom use was only 15% among those not willing to try PrEP. The authors evinced that both PrEP willingness and condom use may be driven by perceived HIV risk. In the study by Falcao et al. [86], the authors found that “anticipated changes in participants’ sexual practices due to uptake of PrEP differed according to participants’ risk perception,” and further, that women tended to perceive higher HIV risk than men [103] and were therefore more likely to insist on continued condom use after PrEP initiation; an observation supported by Rodger et al. [79]. In addition, condom use might persist after PrEP uptake to prevent pregnancy or other STIs [108].

### PrEP and viral suppression (Fig 7)

Evidence indicates that the added protective benefit of PrEP is negligible to none when the HIV-positive partner is virally suppressed [109–111]. This is reflected in provider attitudes and prescription practices denoting some disinclination to prescribe PrEP to HIV-serodifferent couples if the HIV-positive partner is virally suppressed [30, 35, 74, 112, 113]. Yet, at the population level, studies have shown high HIV transmission among HIV-serodifferent couples even when ART is being prescribed, as ART prescription does not always translate into viral suppression [72]. Further, as noted by Gilbert et al., little is known about the perspectives and lived experiences of serodifferent couples themselves with regard to ART and PrEP [68]. We found no conclusive quantitative association between PrEP use and either viral load at last test or sustained viral control in the HIV-positive partner among couples in our study. In qualitative interviews, however, some couples expressed concerns about the lack of durable viral suppression in the HIV-positive partner and indicated that PrEP served as a critical safety net against transmission. Moreover, even some couples achieving sustained viral suppression endorsed concurrent PrEP use. Our finding that some serodifferent couples preferred various forms of combination HIV prevention including both viral suppression and PrEP is consistent with several prior studies [13, 68]. In a qualitative study of HIV-negative partners in serodifferent relationships in Uganda, Gilbert et al. [68] noted distress caused by PrEP discontinuation once the HIV-positive partner had achieved durable viral suppression, resulting in reluctance to engage in condomless sex, especially among the female HIV-negative partners.

These findings may be dismissed simply as a lack of couples’ knowledge regarding the optimal efficacy of TasP (U = U), but expanding evidence suggests that psychosocial, relational, and behavioral context should be considered in couple-based HIV prevention strategies [68, 73]. For example, consistent with prior studies [13, 61, 98, 104, 114], thematic analysis of our qualitative data revealed that many HIV-negative partners desired PrEP for reasons that went beyond their partner’s viral status in order to actively participate in HIV prevention and contribute to the well-being of the couple. As a result, they strengthened their relationship and maintained some autonomous control over their own protection. This latter theme may be related to the observation in several studies that a substantial proportion of HIV-negative partners in HIV-serodifferent heterosexual relationships may be at risk for HIV from external sexual partners [8, 22, 68, 79, 115–121]. These findings underscore the need for tailored approaches to HIV prevention management among HIV-serodifferent couples [122].

### Viral suppression, condoms and PrEP (Fig 8)

Our analysis also revealed an interaction in which consistent condom use moderated the relationship between viral suppression and PrEP uptake. Specifically, PrEP uptake was lowest among couples who used condoms consistently but lacked sustained viral control; whereas PrEP uptake was highest among couples who engaged in consistent condom use and had achieved sustained viral control. PrEP use was also high among couples not using condoms consistently and who lacked viral suppression. Indeed, four groups emerged in our mixed methods analysis: (1) couples using PrEP along with some combination of viral suppression and condoms—typically all three methods (PrEP Plus group); (2) couples relying primarily on PrEP for prevention, characterized by inconsistent condom use and lack of sustained viral suppression (PrEP Only group); (3) couples who embraced U = U and relied mostly on viral suppression and therefore tended to use condoms inconsistently and had lower PrEP use (Viral Control group); (4) couples who relied primarily on condoms for protection and tended to lack sustained viral suppression and had low PrEP uptake (Condom-Centric group)

As noted by Falcao et al. [86] and others [80, 92, 123], this diversity of HIV prevention approaches by HIV-serodifferent couples stems from complex dyadic negotiation and “varying prioritization within different social and individual contexts,” which often involves sex/gender-based dynamics [1, 68, 84, 89, 124]. It also derives from the different ways in which couples conceptualize their HIV “serodiscordance” [125].

PrEP Plus group (n = 8; 30%): Prior studies have demonstrated that love and intimacy in primary relationships often take precedent over health concerns, which may partly explain the observed low use of condoms among HIV-serodifferent couples [3, 126]. However, we found a sizeable group of couples in our cohort who made HIV prevention a priority and strove to use multiple methods for prevention, with PrEP and viral suppression as core strategies, but often also including condom use. PrEP Only group (n = 8; 30%): While all couples endeavored to achieve viral control, when viral suppression could not be sustained, a substantial contingent of couples preferred PrEP as their sole backup method of protection and refrained from using condoms. The advantage of PrEP over condom use was viewed as proffering a “normal” sex life, thereby enhancing relationship intimacy [13, 61, 86, 104]. These first two groups were also described in a qualitative study of 20 HIV-serodifferent couples enrolled in the Partners Demo Project [61]. Viral Control group (n = 4; 15%): Couples in our study who eschewed both condom use and PrEP, and relied solely on viral suppression (mostly couples with male HIV-negative partners) pointed to their long history of condomless sex and lack of HIV transmission as confirmation of the effectiveness of their approach; a finding consistent with prior research [67, 73]. However, most of the HIV-positive females in these relationships wanted their male partners to initiate PrEP for added protection. These couples, especially HIV-negative male partners, tended to avoid discussion of HIV and their “serodiscordance”—what Persson referred to as “sero-silence” [41]. Other barriers to PrEP use in this group included stigma, medication aversion, and compliance challenges, which have been reported in previous studies [98, 105, 127]. Condom Centered group (n = 3; 11%): A few couples in our study (all female HIV-positive) relied solely on condoms for protection. In these relationships, the female partners struggled to maintain viral suppression and tended to be strong advocates for condom use. Thus, the male HIV-negative partners in these couples saw no added advantage of using PrEP if their female partners insisted on continued condom use. The fact that condoms also protect against other sexually transmitted infections and unwanted pregnancy, which PrEP does not, was found in prior studies to be a motivation to use condoms among serodifferent couples, primarily driven by female partners [84, 106, 128].

### HIV transmission anxiety (Fig 9)

Evidence is clear that stress and anxiety play an important role influencing HIV prevention behaviors [76, 88, 129–131]. In addition, several studies have demonstrated an association between high levels of anxiety and lower ART adherence and enhanced disease progression [132–134], although the literature is far from conclusive regarding the mechanisms at play [135, 136]. One type of anxiety that emerged as a recurrent theme in our study of heterosexual serodifferent couples involves anxiety over transmitting HIV to the negative partner. In general, female partners reported higher levels of HIV transmission anxiety than did male partners, a finding consistent with the work of Kennedy and colleagues [137] who, more than 25 years prior, found higher levels of anxiety among the female partners of couples affected by HIV (also see [138]). We also found that HIV-positive partners had greater transmission anxiety than HIV-negative partners, regardless of gender, an observation also noted in the work of Asha Persson [66]. It is therefore not surprising that HIV transmission anxiety was especially pronounced among the female HIV-positive partners in our sample, a finding also reported by Kelly et al. [80] and Carroll et al. [84].

Prior research has shown that knowledge of viral control and treatment-as-prevention can substantially reduce HIV transmission anxiety in serodifferent couples [66]. Yet, although ART adherence and viral suppression were ostensibly under the control of the HIV-positive females in our study, the majority (10/16) reported lack of sustained viral suppression. Bourne et al. [69] similarly described how HIV-positive women in serodifferent relationships felt anxious about relying solely on viral suppression to prevent HIV transmission to their partners (also see [68]). Further, some evidence indicates that greater control and responsibility can sometimes lead to enhanced stress [139]. Given that many HIV-positive females in our study encountered challenges negotiating condom use with their male partners—a finding consistent with prior research [84, 140]–PrEP was viewed as a means of proactively coping with and reducing anxiety around potential HIV transmission. Thus, our findings are consistent with prior research showing that in many serodifferent couples, PrEP was viewed as a potential solution to what has been termed the “discordance dilemma” [104]: the conflict between the need for intimacy (marked by condomless sex) and the need to alleviate anxiety around HIV transmission [13, 80, 88, 98, 106, 141–143].

The HIV-negative males in our sample reported the lowest anxiety around dyadic HIV transmission, despite their relatively low use of condoms (4/16) and PrEP (7/16). In contrast, Falcao et al. [86] and others [24] have found that HIV-negative men in serodifferent heterosexual relationships perceived PrEP use as a way to increase condomless sex and reduce transmission anxiety. The HIV-positive males in our study were mostly supportive of PrEP use by their female partners and also reported slightly higher levels of transmission anxiety compared to HIV-negative males. Similarly, Carroll et al. [84] reported that HIV-positive men in serodifferent heterosexual relationships in Kenya were generally supportive of both condom use and PrEP; and Persson [41] observed “an express effort to protect the women’s HIV-negativity” by HIV-positive men in Australian serodifferent couples. In addition to the observed patterns of HIV transmission anxiety by sex/gender and HIV status, at the dyadic level, Bagheri et al. [144] have shown that higher levels of anxiety in one partner can actuate greater anxiety in the other partner. Our findings, along with prior evidence, highlight the critical influence of transmission anxiety on HIV prevention behaviors among HIV-serodifferent heterosexual couples, which may be moderated by sex/gender and relationship dynamics.

### Limitations

Several limitations are inherent in our study. The cross-sectional study design precludes making definitive causal statements. All data are based on self-report, and thus subject to various forms of response bias, including social desirability and inaccurate recall. Knowledge of treatment-as-prevention and PrEP varied considerably across participants, which may have influenced both the behavior and perspectives of participants with regard to HIV prevention strategies. Indeed, some of the couples who were not on PrEP at the time of the interview later initiated PrEP use, perhaps due to knowledge gained as a result of their participation in the study. Thus, study findings should be interpreted as reflecting couple-based perspectives and behaviors in a particular “slice of time.” Demographically and geographically, study participants were a relatively homogeneous group. Moreover, various forms of sampling bias may also have contributed to sample homogeneity; for example, recruitment of couples was conducted at a limited number of clinical or social service agencies. PrEP costs are generally covered under various New York State programs and were not a barrier for couples, but this may vary by state. In addition, self-selection bias may have occurred due to enrollment of couples who were willing to discuss their HIV-serodifferent status and other sensitive topics. As noted by Persson [145] and others [146], some serodifferent couples practice “sero-silence” in which any discussion of their mixed HIV status is avoided to promote heteronormality, and these couples may have been underrepresented in our sample. In addition, pregnancy intentions can significantly impact HIV prevention strategies, but we were not able to discern themes or associations in this regard given that only three of the couples in the study reported trying to conceive. As a result, our findings may not generalize to HIV-serodifferent heterosexual couples in all contexts or settings. There have been several new advances in HIV prevention (e.g., long-acting injectable ART and PrEP) since data were collected in this study that may affect couples’ prevention practices, but the underlying dynamics driving couple-based strategies have remained unchanged. The sample was also relatively small for a mixed-methods study and power was especially limited for the quantitative results.

## Conclusions

A substantial proportion of heterosexually acquired HIV infections in the U.S. occur between partners in primary relationships characterized by mixed HIV status. Yet, we know little about HIV-serodifferent heterosexual couples’ perspectives, experiences and behaviors regarding combination HIV prevention strategies involving condoms, viral suppression and PrEP. Our mixed methods study, conducted with primarily Black/African American and Latinx mixed-status couples, shed some light on this issue. Most couples were aware of the HIV prevention benefits offered by ART adherence and an undetectable viral load in the HIV-positive partner; however, less than half reported achieving long-term sustained viral suppression, prompting many couples to have apprehensions about relying solely on viral suppression for protection. Less than one-third of couples in our study used condoms consistently and about one-third never used condoms. HIV-negative partners were taking PrEP in a majority of couples (16/27).

We found considerable heterogeneity in the use of HIV prevention methods in this sample, ranging from couples who used no form of protection to couples who used consistent triple protection (condoms, viral suppression and PrEP), and all combinations thereof. Our analysis revealed several notable patterns in HIV prevention strategies adopted by HIV-serodifferent heterosexual couples, and these were largely driven by sex/gender and relationship dynamics. Notably, we found that HIV-positive female partners had the highest level of anxiety around dyadic HIV transmission, and this trepidation was born out by the observation that only 38% reported achieving sustained viral suppression, only 25% of female HIV-positive couples used condoms consistently, and only 44% of male HIV-negative partners were taking PrEP. Female partners tended to view condoms more favorably than males; and couples with HIV-negative female partners were nearly twice as likely to report any condom use compared to couples with male-negative partners. This suggests that HIV-negative status within serodifferent couples may confer some degree of agency within the relationship with regard to condom use behavior.

Given the low levels of condom use and lack of sustained viral suppression among HIV-serodifferent couples, PrEP emerged as a critical prevention method in this group. Although both condom and PrEP use tended to be lower in male HIV-negative couples compared to female HIV-negative couples, there was substantial heterogeneity in PrEP use by serodifferent couples. PrEP use tended to be high among couples who were also using condoms consistently and reported sustained viral control, and these were predominantly female HIV-negative couples. PrEP use was also high among both female and male HIV-negative partners in couples characterized by inconsistent condom use and lack of sustained viral control. The most striking sex/gender-based division in PrEP use was observed among couples who were adherent to at least one other form of HIV prevention method (i.e., either consistent condom use and/or viral suppression). In such couples, PrEP uptake among male HIV-negative partners was low (2/9; 22%); whereas PrEP use among female HIV-negative partners in such couples was high (6/7; 86%).

The contention that HIV-negative partners may have more agency within HIV-serodifferent couples to influence HIV prevention behavior is intriguing and is consistent with our mixed methods findings. When female HIV-positive partners reported sustained viral suppression, most of their male HIV-negative partners did not adopt PrEP and most of these couples were not using condoms consistently. Similarly, none of the male HIV-negative partners were using PrEP if the couple was using condoms consistently, regardless of their partner’s viral load status. In contrast, when male HIV-positive partners reported sustained viral suppression, most of their female HIV-negative partners were using PrEP for additional protection, and many were also using condoms. Thus, male HIV-negative partners were more content to rely on a single HIV prevention method, whereas female HIV-negative partners desired multiple methods of protection.

The adoption of HIV prevention methods by HIV-serodifferent heterosexual couples is complex and shaped in large part by sex/gender and relationship dynamics, as well as social and cultural context [1, 13, 84, 85, 89, 98]. Several implications for healthcare professionals providing HIV prevention counseling to mixed HIV status heterosexual couples emerge from our study, supported by prior research. HIV prevention strategies should be tailored to each couple’s social situation by considering the psychosocial ramifications of different prevention strategies [80]. Anxiety around HIV transmission, impaired relationship quality, and stigma can all have detrimental effects on the health and well-being of members of serodifferent couples [41, 123]. Healthcare providers need to consider the risk of such psychosocial harms in addition to clinical and biomedical considerations [73, 147]. Moreover, the heightened anxiety among both HIV-negative and HIV-positive females in serodifferent relationships in our study is empirically justified. It is known that females are more susceptible to heterosexual transmission of HIV than are males [148, 149] and have historically lacked control over HIV prevention methods [150]. In addition, for females especially, HIV prevention preferences may be informed by conception or contraception intentions [82, 151, 152], as well as the desire for protection against other sexually transmitted infections [153], which may arise from sexual activity by either partner outside the primary relationship [79, 116, 154]. Females in particular may view PrEP, in combination with other HIV prevention methods, as a means of taking control for their own safety and for strengthening their relationship through shared responsibility of HIV prevention. Sexual health counselors should be attentive to such motivations for multiple forms of protection [12, 74].

Given that regular sexual contact introduces a routine risk of HIV transmission, HIV-serodiscordant couples are a key population in which to understand how social and behavioural factors may affect risk perception and management, and inform comprehensive HIV prevention measures [123].
